# A Microplane Constitutive Model for SFRC Subjected to High Temperatures

**DOI:** 10.3390/ma19112229

**Published:** 2026-05-25

**Authors:** Marianela Ripani, Sonia Vrech, Antonio Caggiano, Paula Folino

**Affiliations:** 1 Consejo Nacional de Investigaciones Científicas y Técnicas (CONICET), Buenos Aires C1425FQB, Argentina; mripani@fi.uba.ar (M.R.); svrech@herrera.unt.edu.ar (S.V.); 2 Departamento de Ingeniería, Universidad Nacional del Sur, Bahía Blanca, Buenos Aires 8000, Argentina; 3 Laboratorio de Métodos Numéricos en Ingeniería (LMNI), Facultad de Ingeniería, Universidad de Buenos Aires, Buenos Aires C1127AAR, Argentina; pfolino@fi.uba.ar; 4 Centro de Métodos Numéricos y Computacionales en Ingeniería (CEMNCI), Facultad de Ciencias Exactas e Ingeniería, Universidad Nacional de Tucumán, Tucumán T4000BMD, Argentina; 5 Dipartimento di Ingegneria Civile (DICCA), Chimica e Ambientale, Università degli Studi di Genova, 16145 Genova, Italy; 6 Instituto de Tecnologías y Ciencias de la Ingeniería Hilario Fernández Long (INTECIN), Universidad de Buenos Aires and Consejo Nacional de Investigaciones Científicas y Técnicas, Buenos Aires C1063ACV, Argentina

**Keywords:** concrete, steel fiber, SFRC, high temperature, microplanes, mixture theory, thermodynamic consistency

## Abstract

**Highlights:**

- Thermodynamically consistent microplane model for heated SFRC.
- Temperature-dependent degradation of matrix and fiber interactions.
- Residual constitutive behavior described by crack opening/slip laws.
- Acoustic tensor analysis used to identify bifurcation conditions.
- Failure orientation evaluated for different thermal conditions.

**Abstract:**

Despite the low thermal conductivity that characterizes the mechanical behavior of cementitious composites like concrete, high temperatures acting for long periods could have devastating effects on the overall integrity and stability of structures. Such damage encompasses not only the structural but also the material level, manifested as a degradation of the strength and stiffness properties together with increasing porosity and the consequent cohesion loss. Adding fibers to the cementitious matrix is a strategy that increases the fire resistance of structures, improving the fracture energy release capacity beyond the peak strength. This fact has been experimentally demonstrated in numerous publications and requires the development of advanced computational constitutive models with the aim of predicting the evolution of both elastic properties and failure behavior in fiber-reinforced concrete. In this work, a temperature-dependent, thermodynamically consistent microplane material model based on the smeared crack approach is developed to simulate the mechanical behavior of preheated steel fiber-reinforced concrete (SFRC) under residual conditions. The influence of high temperatures on the material response is evaluated in terms of stress versus crack opening displacement or crack slip curves, whereas the failure analysis in the form of discontinuous bifurcation is addressed by means of numerical analysis of the acoustic tensor, identifying the critical orientation for varying temperature levels, material properties and boundary conditions.

## 1. Introduction

When cementitious composites like concrete are subjected to high temperatures in long-term exposures, two effects are evident. On the one hand, and as a result of the dehydration process, there is an irreversible degradation of two fundamental material properties: the elastic stiffness (thermal damage) and the material strength (thermal decohesion). On the other hand, a particular failure mode develops—spalling, which is characterized by fracture planes parallel to the heated surface and perpendicular to the temperature flux. These events lead to severe degradation of the mechanical properties of cementitious composites and changes in the failure mechanisms of structures [1].

As widely demonstrated by laboratory tests, the incorporation of supplementary cementitious materials, such as silica fume, fly ash, ground granulated blast furnace slag, metakaolin and slag powder, positively influences the performance of cement-based materials at temperatures between 100 °C and 700 °C. Similarly, the addition of specific fibers enhances particular material properties [2]. Some fibers exhibit low thermal stability, such as Polypropylene, PVA (Polyvinyl alcohol) and PE (Polyethylene). They melt at relatively low temperatures, providing pathways for water vaporization within the cementitious matrix. Consequently, the internal vapor pressure is reduced, leading to improved spalling resistance [3,4,5]. Steel, carbon and basalt fibers exhibit thermal stability and good mechanical properties at both room temperature and high temperatures. They can therefore provide resistance during and after fire by restricting the initiation and propagation of microcracks through the bridging effect [6,7,8,9].

Indeed, the addition of steel fibers strongly contributes to preventing the risk of explosive spalling more effectively, improving ductility in the post-cracking phase and reducing the potential brittle failure mode across all laboratory tests, including the following tests:

- Uniaxial tension: SFRC exhibits higher residual tensile strength than plain concrete. In addition, structural integrity is preserved because fibers restrict crack growth, leading to more distributed damage [10,11].
- Bending: the addition of fibers to the cementitious matrix improves the bending stiffness of concrete and clearly contributes to mitigating the mechanical degradation induced by high temperature [11,12,13,14].
- Direct shear: steel fibers improve ductility and higher shear strength at elevated temperatures, mainly through their influence on contact friction, dilatancy and cohesion. The residual strength is therefore assumed to be mainly governed by contact friction [4,15,16].
- Uniaxial compression: the addition of fibers does not lead to a significant increase in compressive strength; however, any beneficial effects on mechanical behavior (such as improved ductility or post-peak response) tend to be preserved even at high temperatures [12,17,18,19].
- Triaxial compression: enhanced peak and residual triaxial compressive strengths are observed after exposures to temperatures between 200 °C and 400 °C, whereas a decrease occurs after exposure above 400 °C. Failure modes also vary with the level of confinement: for certain combinations of confinement and temperature, shear failure is observed, whereas at higher temperatures no evident surface cracking is detected [20].

Finally, increasing temperature has been shown to adversely affect the pull-out behavior of steel fibers from the concrete matrix and, consequently, their contribution to the residual mechanical response [19,21]. In addition, both concrete mass loss and steel fiber ductility increase with temperature.

Beyond experimental observations, temperature-dependent mechanical properties of SFRC, in terms of relative residual elastic modulus and uniaxial compressive and tensile strengths have also been numerically described by means of empirical regression equations, such as those proposed by Zheng et al. [22], Zheng et al. [9] and Zheng et al. [23]. However such approaches remain essentially phenomenological and do not explicitly represent the underlying physical mechanisms. To overcome this limitation, several constitutive models grounded in well-established material theories have been developed at different observation scales:

- At the macroscopic level, where matrix and reinforcements are not differentiated, constitutive models are based on elasto-plastic [24,25] and damage theories [26], as well as on data-driven and machine learning approaches that correlate mechanical attributes with structural performance [27,28].
- At the mesoscopic level, the heterogeneous nature of concrete makes this scale particularly suitable for capturing the interaction between its constituents. In this context, the material is typically described as a three-phase composite consisting of aggregates, mortar matrix and interfacial transition zones. This approach has been successfully applied, for instance, to geopolymer concrete by Shi et al. [29], and to fiber-reinforced concrete by Zhang et al. [30]. However, in most cases, the relative slip between fibers and matrix is neglected.
- At the micromechanical level, material models are able to predict macroscopic responses, but they are based on the simulation of single-fiber pull-out mechanisms [31].

In view of these existing approaches, a macroscopic constitutive model that incorporates relevant micro- and mesoscale mechanisms being thermodynamically consistent and computationally efficient appears particularly promising. In this work, the thermodynamically consistent microplane framework proposed by Carol et al. [32] and Kuhl et al. [33] is adopted and systematically extended to account for temperature-dependent behavior and to incorporate fiber reinforcement through the mixture theory, leading to a novel constitutive model for simulating the failure behavior of preheated SFRC under residual conditions. Mixture Theory is employed to describe fiber–matrix interaction mechanisms, namely axial bond–slip and dowel effects. An additional advantage of the microplane formulation is the possibility of defining one-dimensional failure functions for each material phase at the microplane level, together with a relatively simple computational implementation. Within this framework, the thermodynamically consistent constitutive model for quasi-brittle cementitious materials proposed by Vrech et al. [34] is extended here by introducing temperature dependence into the elasto-plastic formulations describing the bond–slip and dowel mechanisms of fibers crossing active cracks. In addition, the proposed formulation incorporates degradation of the fiber–matrix interface, together with degradation of the elastic properties of the cementitious matrix itself induced by temperature effects.

After this introduction, Section 2 extends the thermodynamic consistency of the microplane theory applied to SFRC at elevated temperatures. Section 3 introduces the mean expressions of the diffuse and localized failure analysis for microplane elasto-plasticity. Section 4 describes the application of mixture theory to account for the contribution of each constituent to the overall SFRC behavior. Section 5 presents the temperature-dependent failure criterion, loading surfaces, plastic potential and elastic properties evolution of the cementitious matrix, while Section 6 summarizes the constitutive description of crack-bridging mechanisms regarding temperature effects. Numerical results for uniaxial tension and direct shear tests on preheated specimens under residual conditions are reported in Section 7, where they are compared against available experimental data. In addition, localized failure analysis is also carried out to define critical failure directions. Finally, Section 8 presents the main conclusions.

## 2. Thermodynamic Consistency of Elasto-Plastic Microplane Theory Under High Temperature

Although the general framework of thermodynamic consistency of the elasto-plastic Microplane Theory has been well established by Carol et al. [32] and Kuhl et al. [33], its extension to account for temperature dependence within the present formulation constitutes a specific contribution of this work.

**Figure 1:**
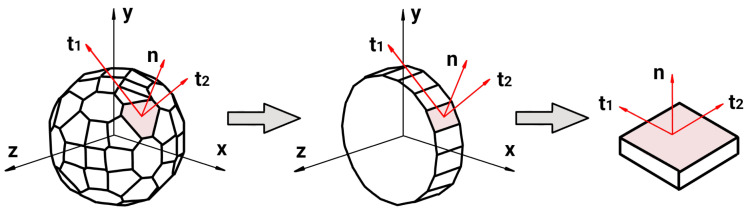
Microplane adopted configurations.

As is well known, under the assumption of kinematic constraints, microplane theory assumes that each material point can be represented by a unit sphere discretized into a finite number $n_{mp}$ of microplanes, with each one identified by its normal direction ***n***; see Figure 1.

This approach provides a directional framework in which the macroscopic strain tensor is projected into components associated with each microplane orientation. The constitutive behavior is then formulated at the microplane level in terms of these components, and the corresponding microplane stresses are later integrated over all orientations to recover the macroscopic response. Under plane stress or plane strain conditions, the microplane integration over the unit sphere can be consistently reduced to an integration over a unit circle, since out-of-plane orientations do not provide independent kinematic or static information. Therefore, a two-dimensional microplane discretization is sufficient to fully represent the constitutive response [35]. This concept is schematically illustrated in Figure 1.

By projecting the total strain tensor ε onto the microplanes, the normal and tangential micro-strains are obtained as

(1) $$\begin{array}{cc} \varepsilon_{N} & =N:\varepsilon ,\;N=n⊗n\\ \varepsilon_{T} & =T:\varepsilon ,\;T=n\cdot I^{sym}-n⊗n⊗n\;, \end{array}$$

where ***N*** and ***T*** denote the normal and tangential projection tensors, respectively, and $I^{sym}$ represents the symmetric part of the fourth-order identity tensor. Thus, the tangential strain is given by $\varepsilon_{T}=\left[\varepsilon_{T1},\;\varepsilon_{T2}\right]$; see Figure 1.

To account for dissipative elasto-plastic behavior, the strain components are further decomposed into elastic and plastic parts following the Prandtl–Reuss framework, i.e., $\varepsilon_{N}=\varepsilon_{N}^{e}+\varepsilon_{N}^{p}\;and\;\varepsilon_{T}=\varepsilon_{T}^{e}+\varepsilon_{T}^{p}$.

The proposed formulation is now established within the framework of continuum thermodynamics. In particular, the response on each microplane is characterized by a suitable free-energy potential, while the admissibility of the constitutive equations is enforced through the Clausius–Duhem inequality. The micro free-energy density $\psi^{mic}$ is defined in a decoupled form by the sum of elastic and inelastic contributions, named $\psi^{mic,e}$ and $\psi^{mic,p}$, respectively. The elastic component is expressed in terms of the elastic strain projections, whereas the inelastic one depends on an internal variable κ, taken as a scalar in the case of isotropic hardening/softening behavior. Moreover, both counterparts are assumed to be temperature-dependent, leading to

(2) $$\psi^{mic}\left(\varepsilon_{N}^{e},\varepsilon_{T}^{e},\kappa ,T\right)=\psi^{mic,e}\left(\varepsilon_{N}^{e},\varepsilon_{T}^{e},T\right)+\psi^{mic,p}\left(\kappa ,T\right)\;.$$

Based on the defined free-energy potential, the thermodynamic restrictions on the constitutive behavior can be established by considering the corresponding Clausius–Duhem dissipation inequality at the microplane level, which reads

(3) $$\sigma_{N}{\dot{\varepsilon }}_{N}+\sigma_{T}\cdot {\dot{\varepsilon }}_{T}-\rho {\dot{\psi }}^{mic}-\rho \dot{T}s^{mic}-\frac{h\cdot \nabla T}{T}\geq 0\;,$$

where ρ denotes the mass density, $s^{mic}$ the entropy, ***h*** the heat flux vector and ∇T the temperature gradient.

By enforcing the Clausius–Duhem inequality in Equation (3) in conjunction with the Coleman procedure, the constitutive relations for the normal and tangential stresses $\sigma_{N}$ and $\sigma_{T}$, respectively, as well as the associated thermodynamic dissipative stresses $\phi^{mic}$ and entropy $s^{mic}$ are obtained by differentiating $\psi^{mic}$ with respect to the corresponding conjugate variables and temperature, respectively, as follows

(4) $$\begin{array}{cc} \sigma_{N}\left(\varepsilon_{N}^{e},T\right) & =\frac{\partial \psi^{mic}}{\partial \varepsilon_{N}^{e}}\;,\;\sigma_{T}\left(\varepsilon_{T}^{e},T\right)=\frac{\partial \psi^{mic}}{\partial \varepsilon_{T}^{e}}\\ \phi^{mic}\left(\kappa ,T\right) & =-\frac{\partial \psi^{mic}}{\partial \kappa }\;,\;s^{mic}\left(T\right)=-\frac{\partial \psi^{mic}}{\partial T}\;. \end{array}$$

The corresponding mechanical and thermal dissipation rates at the microplane level, $D^{mic,m}$ and $D^{mic,th}$, respectively, must satisfy the following conditions

(5) $$D^{mic,m}\left(\kappa ,T\right)=-\frac{\partial \psi^{mic}}{\partial \kappa }\dot{\kappa }\geq 0\;and\;D^{mic,th}\left(T\right)=-\frac{h\cdot \nabla T}{T}\geq 0\;.$$

Due to the dependence of $\psi^{mic}$ on the temperature field, the mechanical dissipation is also influenced by T. On the other hand, the thermal dissipation vanishes under isothermal conditions, i.e., in the absence of temperature gradients.

A particular form of the elastic component of the free-energy potential in Equation (2) for a specific material such as concrete yields

(6) $$\psi^{mic,e}\left(\varepsilon_{N}^{e},\varepsilon_{T}^{e},T\right)=\frac{1}{2}E_{N}\left(T\right)\;{\varepsilon_{N}^{e}}^{2}+\frac{1}{2}E_{T}\left(T\right)\;\varepsilon_{T}^{e}\cdot \varepsilon_{T}^{e}-\frac{1}{2}XT^{2}-E_{N}\left(T\right)\;T\;\zeta_{N}\;\varepsilon_{N}^{e}-E_{T}\left(T\right)\;T\;\zeta_{T}\;\varepsilon_{T}^{e}\;,$$

where X denotes the thermal capacitance, while $\zeta_{N}$ and $\zeta_{T}$ represent the directional thermal expansion coefficients. The first and second terms of Equation (6) correspond to the normal and tangential elastic stored energy, governed by the temperature-dependent stiffnesses $E_{N}\left(T\right)$ and $E_{T}\left(T\right)$, respectively. The third term represents the purely thermal contribution, independent of the mechanical strains. The last two account for thermo-mechanical coupling effects, where the temperature interacts with the normal and tangential elastic strains through the coefficients $\zeta_{N}$ and $\zeta_{T}$, thus incorporating thermal expansion effects at the microplane scale.

The plastic contribution of $\psi^{mic}$ is defined as the substraction of mechanical and thermal terms, given by

(7) $$\psi^{mic,p}\left(\kappa ,T\right)=\frac{1}{2}H^{mic}\left(T\right)\kappa^{2}-TS_{fr}^{mic}\left(\kappa \right)\;,$$

being $H^{mic}\left(T\right)$ the hardening/softening function also influenced by temperature and $S_{fr}^{mic}\left(\kappa \right)$, the frozen entropy proposed by [36].

Substituting Equations (6) and (7) into the constitutive relations of Equations (4) yields the explicit expressions

(8) $$\begin{array}{cc} \sigma_{N} & =E_{N}\left(T\right)\;\left(\varepsilon_{N}^{e}-\zeta_{N}\;T\right)\;,\;\sigma_{T}=E_{T}\left(T\right)\;\left(\varepsilon_{T}^{e}-\zeta_{T}\;T\right)\\ \phi^{mic} & =-H^{mic}\left(T\right)\;\kappa +T\frac{\partial S_{fr}^{mic}}{\partial \kappa }\;,\;s^{mic}=-\frac{\partial \psi^{mic,e}}{\partial T}+S_{fr}^{mic}+\frac{\kappa^{2}}{2}\frac{\partial H^{mic}\left(T\right)}{\partial T}\;. \end{array}$$

According to the classical plasticity flow theory, the evolution laws for the plastic strain components and the internal variable, defined in terms of convex yield function Φ and plastic potential $\Phi^{*}$, are given by

(9) $${\dot{\varepsilon }}_{N}^{p}=\dot{\lambda }\frac{\partial \Phi^{*}}{\partial \varepsilon_{N}}\;,\;{\dot{\varepsilon }}_{T}^{p}=\dot{\lambda }\frac{\partial \Phi^{*}}{\partial \varepsilon_{T}}\;,\;\dot{\kappa }=\dot{\lambda }\frac{\partial \Phi^{*}}{\partial \kappa }\;,$$

with $\dot{\lambda }$ being the non-negative plastic multiplier parameter. The classical Kuhn–Tucker loading/unloading and the consistency conditions must be also considered: $\Phi \leq 0,\;\dot{\lambda }\geq 0,\;\Phi \dot{\lambda }=0,\;\dot{\Phi }\dot{\lambda }=0$. Based on the last one and according to the following nomenclature

(10) $$\begin{array}{cc} n_{N} & =\partial \Phi /\partial \sigma_{N}\;,\;n_{T}=\partial \Phi /\partial \sigma_{T}\\ m_{N} & =\partial \Phi^{*}/\partial \sigma_{N}\;,\;m_{T}=\partial \Phi^{*}/\partial \sigma_{T}\;, \end{array}$$

the expression of $\dot{\lambda }$ can be deduced as

(11) $$\dot{\lambda }=\frac{E_{N}\left(T\right)n_{N}{\dot{\varepsilon }}_{N}+E_{T}\left(T\right)n_{T}\cdot {\dot{\varepsilon }}_{T}+\left[\frac{\partial \Phi }{\partial T}-E_{N}\left(T\right)n_{N}\zeta_{N}-E_{T}\left(T\right)n_{T}\cdot \zeta_{T}\right]\dot{T}}{h^{mic}\left(T\right)-H^{mic}\left(T\right)}\;,$$

being

(12) $$h^{mic}\left(T\right)=E_{N}\left(T\right)\;n_{N}m_{N}+E_{T}\left(T\right)\;n_{T}\cdot m_{T}\;.$$

This expression indicates that, due to the thermal degradation of the elastic parameters, the dissipative response is also affected by the temperature field.

Having defined the constitutive behavior at the microplane level, the formulation can be homogenized to obtain the macroscopic response, where the free-energy potential ($\psi^{mac}$) is obtained as the integral of the microplane contribution over the unit sphere Ω, i.e.,

(13) $$\psi^{mac}=\frac{3}{4\pi }\int_{\Omega }\psi^{mic}d\Omega \;,$$

as proposed by Carol et al. [32]. Accordingly, the macroscopic stress tensor σ is given by

(14) $$\sigma =\frac{\partial \psi^{mac}}{\partial \varepsilon^{e}}=\frac{3}{4\pi }\int_{\Omega }N\sigma_{N}+T^{T}\cdot \sigma_{T}\;d\Omega \;,$$

usually solved by a weighted sum over a finite number of microplanes $n_{mp}$, as proposed by Bažant and Oh [37], resulting in

(15) $$\sigma \approx \sum_{I=1}^{n_{mp}}\left[N^{I}{\sigma_{N}}^{I}+T^{T,I}\cdot \sigma_{T}^{I}\right]w^{I}\;,$$

where $w^{I}$ represent the corresponding weight coefficients.

From a macroscopic standpoint, it is convenient to express the constitutive relation describing the elasto-plastic response under high temperature in the form

(16) $$\dot{\sigma }=E^{ep}\left(T\right):\dot{\varepsilon }-E^{T}\dot{T}\;,$$

Therefore, substituting Equation (8) in Equation (14) and after applying certain algebra, it becomes possible to compute the elasto-plastic tangent tensor $E^{ep}\left(T\right)$ in terms of the micro values, as

(17) $$E^{ep}\left(T\right)=E^{e}-\frac{3}{4\pi }\int_{\Omega }\left[E_{N}\left(T\right)m_{N}N+E_{T}\left(T\right)m_{T}\cdot T\right]\frac{\left[E_{N}\left(T\right)n_{N}N+E_{T}\left(T\right)n_{T}\cdot T\right]}{h^{mic}\left(T\right)-H^{mic}\left(T\right)}d\Omega \;,$$

with the elastic component, $E^{e}\left(T\right)$ calculated as

(18) $$E^{e}\left(T\right)=\frac{3}{4\pi }\int_{\Omega }\left[E_{N}\left(T\right)NN+E_{T}\left(T\right)T\cdot T\right]d\Omega \;,$$

and the thermal one, $E^{T}\left(T\right)$ as

(19) $$\begin{array}{cc} E^{T}\left(T\right) & =\frac{3}{4\pi }\int_{\Omega }\left[E_{N}\left(T\right)\zeta_{N}N+E_{T}\left(T\right)\zeta_{T}\cdot T\right]+\\ \; & \;+\left[E_{N}\left(T\right)\;m_{N}N+E_{T}\left(T\right)\;m_{T}\cdot T\right]\frac{\left[\partial \Phi /\partial T-E_{N}\left(T\right)n_{N}\zeta_{N}-E_{T}\left(T\right)n_{T}\cdot \zeta_{T}\right]}{h^{mic}\left(T\right)-H^{mic}\left(T\right)}d\Omega \;. \end{array}$$

For the special case of residual response after exposure to high temperatures, isothermal conditions with constant temperature and no thermal gradients within the material are assumed. In this case, temperature acts as a fixed parameter governing the residual degraded mechanical properties. Accordingly, the free-energy potential of Equations (6) and (7) reduce to

(20) $$\psi^{mic,e}\left(\varepsilon_{N}^{e},\varepsilon_{T}^{e},T\right)=\frac{1}{2}E_{N}\left(T\right)\;{\varepsilon_{N}^{e}}^{2}+\frac{1}{2}E_{T}\left(T\right)\;\varepsilon_{T}^{e}\cdot \varepsilon_{T}^{e}$$

and

(21) $$\psi^{mic,p}\left(\kappa ,T\right)=\frac{1}{2}H^{mic}\left(T\right)\kappa^{2},$$

respectively. Thus, the constitutive equations become

(22) $$\begin{array}{cc} \sigma_{N} & =E_{N}\left(T\right)\;\varepsilon_{N}^{e}\;,\;\sigma_{T}=E_{T}\left(T\right)\;\varepsilon_{T}^{e}\\ \phi^{mic} & =-H^{mic}\left(T\right)\;\kappa \;,\;s^{mic}=-\frac{\partial \psi^{mic,e}}{\partial T}+\frac{\kappa^{2}}{2}\frac{\partial H^{mic}\left(T\right)}{\partial T}\;, \end{array}$$

while the plastic multiplier reduces to the form

(23) $$\dot{\lambda }=\frac{E_{N}\left(T\right)n_{N}{\dot{\varepsilon }}_{N}+E_{T}\left(T\right)n_{T}\cdot {\dot{\varepsilon }}_{T}}{h^{mic}\left(T\right)-H^{mic}\left(T\right)}\;,$$

and the macroscopic elasto-plastic response reduces to

(24) $$\dot{\sigma }=E^{ep}\left(T\right):\dot{\varepsilon }\;.$$

## 3. Microplane-Based Localized Failure Analysis

Within the framework of continuum mechanics, two different failure modes can be distinguished as the load increases: diffuse and localized. In both cases, the displacement field remains continuous $\left([\left[\dot{u}\right]]=0\right)$, whereas the localized failure is characterized by the development of strain discontinuities $\left([\left[\dot{\varepsilon }\right]]\geq 0\right)$.

### 3.1. Diffuse Failure

The mathematical indicator for diffuse failure is adopted as the classical instability criterion proposed by Hill [38], based on the second-order work density (energetic delimiter) $d^{2}W=0$, which leads to the stationary stress condition $\dot{t}=0$. This requirement implies the singularity of the elasto-plastic tangent material tensor, i.e., $\det\left(E^{ep}\right)=0$.

### 3.2. Localized Failure

Localized failure modes are associated with discontinuous bifurcations of the equilibrium path and with the loss of ellipticity of the governing equilibrium equations. In the framework of the smeared crack approach, discontinuity surfaces with normal direction $N_{l}$ are identified by jumps in the strain field, while the displacement field remains continuous. This discontinuity can be numerically detected by solving the eigenvalue problem associated with the acoustic (or localization) tensor; see Ottosen and Runesson [39]:

(25) $$\det\left(Q^{ep}\right)=0\;,$$

where $Q^{ep}$ denotes the elasto-plastic localization tensor defined as

(26) $$Q^{ep}=N_{l}\cdot E^{ep}\cdot N_{l}\;.$$

In the case of materials exposed to high-temperature fields, $Q^{ep}$ also becomes dependent on T, as $E^{ep}\left(T\right)$. This effect will be analyzed in Section 7.

## 4. Mixture Theory for Composite Constitutive Formulation

As proposed by Oliver et al. [40] for reinforced concrete, the constitutive description of SFRC at each microplane involves a composite material formed by three phases: a cementitious matrix and two fiber–matrix interaction mechanisms, namely axial bond–slip and dowel effects.

Following the kinematic framework of mixture theory [41,42,43], where each infinitesimal volume is simultaneously occupied by all constituents and the kinematic field of the equivalent continuum agrees with that of each constituent, the stress vector on each microplane $t=[t_{N}\;t_{T}]$ is assumed to be the weighted sum of the contributions of the matrix and the fiber bridging mechanisms, as

(27) $$t=\omega^{m}\sigma^{m}+\sum_{f=0}^{n^{f}}\omega^{f}\left[\sigma_{N}^{f}n+\sigma_{T}^{f}\cdot n_{T}\right]\;,$$

where the matrix stress vector is computed as $\sigma^{m}=\left[\sigma_{N}^{m}\;\sigma_{T}^{m}\right]$, while the fiber bond–slip and dowel stresses are represented by $\sigma_{N}^{f}$ and $\sigma_{T}^{f}$ according to their normal and tangential directions, ***n*** and $n_{T}$, respectively.

In the case of uniform fiber distribution, the total number of fibers crossing each microplane, denoted as $n^{f}$, can be estimated following the proposals by Krenchel [44] and Dupont and Vandewalle [45] as

(28) $$n^{f}=\alpha_{N}\frac{\omega^{f}}{A^{f}}A^{i}\;,$$

where $\alpha_{N}$ represents the orientation factor (assumed as 0.405 according to Soroushian and Lee [46]), while $A^{f}$ and $A^{i}$ correspond to the single fiber and interface cross-sectional areas, respectively.

At the macroscopic level, the composite material stress tensor σ is computed as

(29) $$\sigma \approx \sum_{I=1}^{n_{mp}}\left[{t_{N}}^{I}{\left(n⊗n\right)}^{I}+t_{T}^{I}\cdot {\left(n_{T}⊗n\right)}^{I}\right]w^{I}\;.$$

## 5. Constitutive Model for the Cementitious Matrix Under High Temperature

The temperature-dependent constitutive formulation for the cementitious matrix at microplane level is developed by extending the one proposed by Vrech et al. [34], originally formulated for plain concrete at ambient temperature. In the present work, the temperature dependency is incorporated into the expressions of the elastic properties, failure criterion, loading surfaces and plastic potential.

### 5.1. Temperature-Dependent Elastic Properties

Given the decoupled nature of the present constitutive formulation, the normal and tangential stress components are conjugated to the corresponding micro-strains through the elastic normal and tangential micro-moduli, $E_{N}$ and $E_{T}$, defined according to Leukart [47] as

(30) $$\begin{array}{cc} E_{N} & =3K\;\mathrm{and}\;E_{T}=\frac{10}{3}G-2K\;,\\ \;\mathrm{with}\;K & =\frac{E}{3(1-2\nu )}\;\mathrm{and}\;G=\frac{E}{2(1+\nu )}\;, \end{array}$$

being *K* and *G* the bulk and shear macroscopic moduli, respectively.

The degradation of elastic properties induced by high temperatures is mathematically reproduced through reduction functions, such as those proposed by Ripani et al. [48] for Young’s modulus *E* and Poisson ratio ν, which are based on an extensive experimental database, as

(31) $$\begin{array}{cc} E(T) & =E_{20}\left(1-\alpha_{E}\;T\right)\;, \end{array}$$

(32) $$\begin{array}{cc} \nu (T) & =\nu_{20}\left(1-\alpha_{\nu }\;T\right)\;, \end{array}$$

where $E_{20}$ and $\nu_{20}$ are the corresponding values at room temperature, whereas $\alpha_{E}$ and $\alpha_{\nu }$ are the degradation parameters.

### 5.2. Failure Criterion

**Figure 2:**
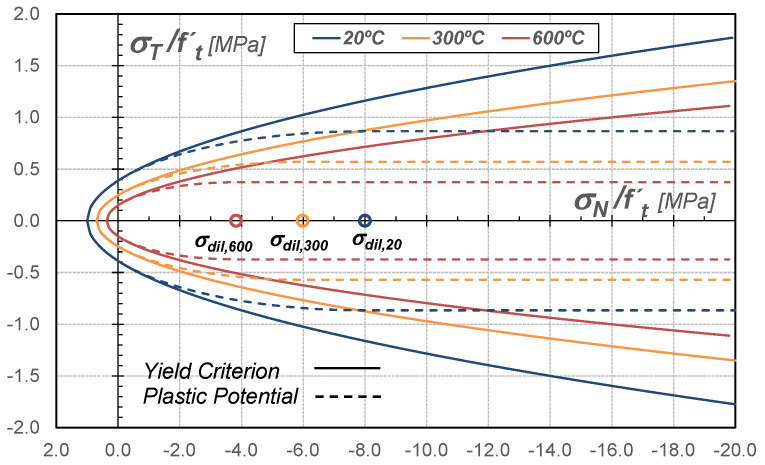
Microplane failure criteria and plastic potentials with increasing temperature. Solid lines indicate the yield criterion, whereas dashed lines represent the plastic potential.

The failure criterion consists of a parabolic function defined in terms of the normal and tangential micro-stresses, $\sigma_{N}^{m}$ and $\sigma_{T}^{m}$, as

(33) $$\Phi^{m}\left(\sigma_{N}^{m},\sigma_{T}^{m},T\right)=\alpha \left(T\right)\left[\frac{{\left(f_{c}^{\prime }+f_{t}^{\prime }\right)}^{2}}{8f_{t}^{\prime }f_{c}^{\prime }}\right]{∥\sigma_{T}^{m}∥}^{2}+\sigma_{N}^{m}-\beta \left(T\right)f_{t}^{\prime }=0\;,$$

where $f_{t}^{\prime }$ and $f_{c}^{\prime }$ denote the uniaxial tensile and compressive strengths, respectively. In Equation (33), the temperature T (in °C) degrades both the residual strengths and the comparison stresses through the temperature-dependent functions α(T) and β(T), defined as

(34) $$\begin{array}{cc} \alpha (T) & =1+\gamma_{1}\left(T-20\right)\;, \end{array}$$

(35) $$\begin{array}{cc} \beta (T) & =1-\gamma_{2}\left(T-20\right)\;, \end{array}$$

with $\gamma_{1}$ and $\gamma_{2}$, temperature-dependent parameters calibrated according to material properties. Figure 2 demonstrates the degradation of the failure functions with increasing temperatures, from 20 to 600 °C.

### 5.3. Loading Surfaces

Once the peak strength has been attained, the post-peak regime is activated and the yield criterion undergoes softening according to

(36) $$\Phi_{s}^{m}\left(\sigma_{N}^{m},\sigma_{T}^{m},\phi^{m},T\right)=\alpha \left(T\right)\left[\frac{{\left(f_{c}^{\prime }+f_{t}^{\prime }\right)}^{2}}{8f_{t}^{\prime }f_{c}^{\prime }}\right]{∥\sigma_{T}^{m}∥}^{2}+\sigma_{N}^{m}-\beta \left(T\right)\phi^{m}=0\;.$$

Within the framework of fracture energy theory, the evolution of the dissipative stress ${\dot{\phi }}^{m}$, which governs the post-peak softening response, is defined in terms of the rate of the internal variable $\dot{\kappa }$ and the temperature T, as

(37) $${\dot{\phi }}^{m}\left(\dot{\kappa },T\right)=f_{t}^{\prime }\;\exp\left(\frac{-5\;h_{T}\left(T\right)}{u_{r}\;G_{I}\left(T\right)}\dot{\kappa }\right)\;,$$

where $u_{r}$ denotes the maximum crack opening displacement, while $h_{T}\left(T\right)$ and $G_{I}\left(T\right)$ represent the temperature-dependent characteristic length and the Mode I fracture energy, respectively. These functions are defined by

(38) $$h_{T}\left(T\right)=h\exp\left[-A_{h}\left(T-20\right)\right]\;,$$

(39) $$G_{I}\left(T\right)=G_{I}\left[A_{G}\;T^{2}+B_{G}\;T+C_{G}\right]\;,$$

where *h* and $G_{I}$ denote the same variables at the reference temperature of 20 °C, respectively. The coefficients in Equations (38) and (39) are calibrated from experimental data.

As the temperature increases, the characteristic length $h_{T}\left(T\right)$ decreases significantly, reaching approximately 17% of its reference value at 600 °C. This reduction dominates over the comparatively mild variation in the fracture energy $G_{I}\left(T\right)$, leading to an accelerated evolution of the dissipative stress $\phi^{m}$. Consequently, the loading surface contracts more rapidly in the stress space, resulting in a more brittle post-peak response and a reduced load-carrying capacity.

### 5.4. Plastic Potential

The definition of a plastic potential ensures a realistic transition from tensile cracking and low-confinement regimes to high-confinement compression regimes. For quasi-brittle materials such as concrete, it is essential to consider dilatancy, which manifests as increasing lateral strain with increasing axial compression. As is well known, at room temperature, dilatancy decreases with increasing confinement and eventually disappears at very high confinement levels. The confinement level at which dilatancy vanishes is denoted by $\sigma_{dil,T}$ [49,50]. As temperature increases, $\sigma_{dil,T}$ takes lower values due to the significant increase in the lateral strains, as demonstrated by Xargay et al. [11]. In addition, the higher thermal expansion of high-strength concrete (HSC) compared with normal-strength concrete (NSC) at temperatures above 400 °C may be attributed to the formation of a greater number of cracks [51].

Regarding the graphical representation of the plastic potential in the stress space defined by the coordinates $\sigma_{N}$-$\sigma_{T}$, the dilatancy angle is formed by the outward normal direction to the plastic potential and the vertical (shear) axis. Directions parallel to the vertical axis correspond to zero dilatancy; see Figure 2. In this work, the proposed plastic flow rule encompasses three well-differentiated regions:

– Mode I fracture and tensile regimen ($\sigma_{N}^{m}\geq$ 0): associated plastic flow,
  (40) $$\Phi^{m*}=\Phi^{m}\;.$$
– Mode II fracture and low-confinement regime ($0>\sigma_{N}^{m}\geq \sigma_{dil}\left(T\right)$): volumetric non-associated plastic flow,
  (41) $$\Phi^{m*}=\Phi^{m}-\frac{\sigma_{N}^{m2}}{2\;\sigma_{dil}\left(T\right)}=0\;.$$
– Medium- and high-confinement regime ($\sigma_{N}^{m}\leq \sigma_{dil}\left(T\right)$): non-associated plastic flow in the absence of dilatancy,
  (42) $$\Phi^{m*}=\Phi^{m}-\sigma_{N}^{m}+\frac{\sigma_{dil}\left(T\right)}{2}=0\;.$$

## 6. Constitutive Temperature-Dependent Models for Crack-Bridging Effects

In this section, the effects of high temperatures on the crack-bridging behavior of steel fibers crossing cracks are considered through both bond–slip and dowel contributions, following the formulation proposed by Vrech et al. [34] for room temperature. Table 1 summarizes the main constitutive laws.

### 6.1. Effect of High Temperature on the Steel Fiber Pull-Out Mechanism

The axial elasto-plastic relationship is defined in terms of the bond–slip elastic modulus $E^{f}\left(T\right)$ and the Prandtl–Reuss additive decomposition of the normal strain expressed as $\varepsilon_{N}^{f}=\varepsilon_{N}^{f,e}+\varepsilon_{N}^{f,p}$, as summarized in the second column of Table 1.

**Figure 3:**
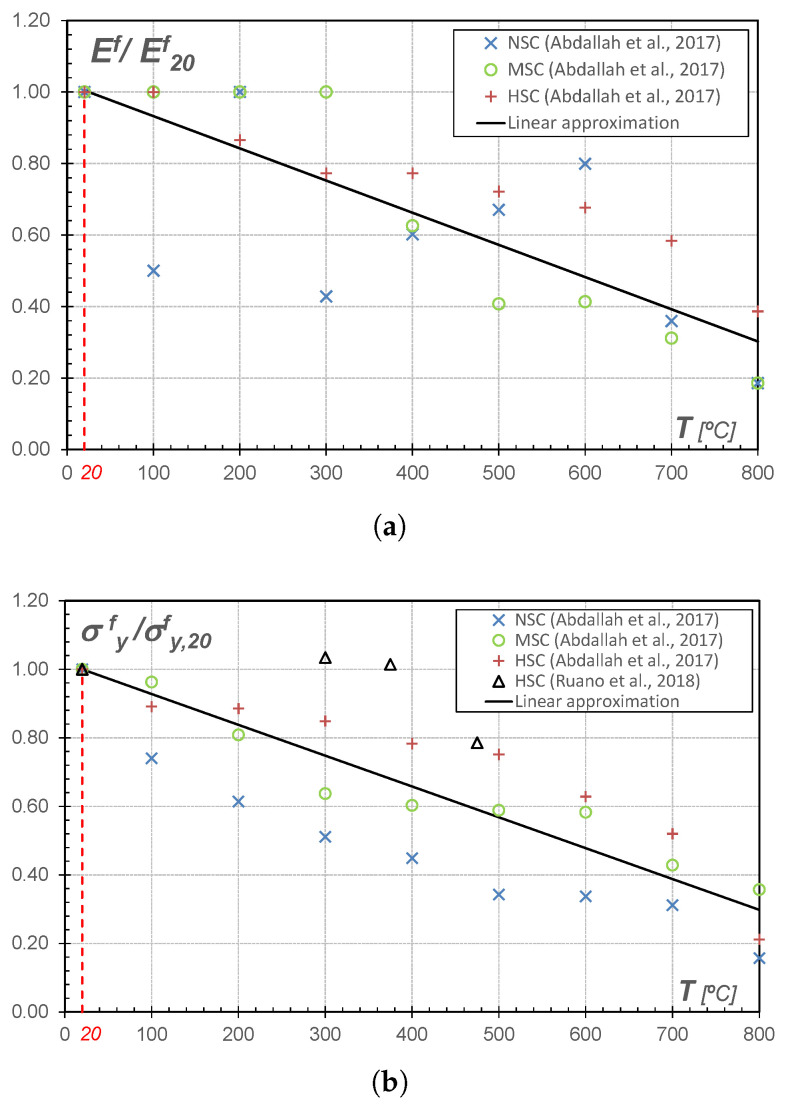
Thermal degradation and normalized approximation functions of (**a**) Bond–slip elastic modulus $E^{f}$ and (**b**) Pull-out strength for steel fibers $\sigma_{y}^{f}$, based on the experimental data reported by [19,21].

As experimentally observed by Abdallah et al. [21], the degradation of the elastic modulus becomes evident as the temperature increases and it is strongly related to the fiber type (straight or hooked-end) and to the quality of the concrete matrix. Considering different matrix qualities and focusing on hooked-end fibers, which are the most commonly used, a linear estimation for the normalized elastic modulus is proposed as

(43) $$\frac{E^{f}\left(T\right)}{E_{20}^{f}}=A_{E}\;T+B_{E}\;,$$

where $E_{20}^{f}$ represents the one at room temperature. The coefficients in Equation (43) are obtained by calibration regarding experimental data. Figure 3a shows good agreement between the experimental results and the numerical predictions.

According to the Rankine-type yield condition, the comparison stress is composed of the axial equivalent fiber yield strength $\sigma_{y}^{f}\left(T\right)$ and the dissipative stress $\phi_{N}^{f}$. In order to incorporate the degradation of $\sigma_{y}^{f}\left(T\right)$ due to the thermal damage of interface and fibers, the experimental results by Abdallah et al. [21] and Ruano et al. [19] were considered. These studies provide load–displacement curves obtained from pull-out tests of hooked-end fibers, validating that the reduction in the pull-out strength begins at a higher temperature than the reduction of the compressive one corresponding to the matrix. For the particular case of hooked-end fibers embedded in the HSC matrix, the equivalent fiber strength can be approximated by the following normalized linear function

(44) $$\frac{\sigma_{y}^{f}\left(T\right)}{\sigma_{y,20}^{f}}=A_{\sigma }\;T+B_{\sigma }\;,$$

where $\sigma_{y,20}^{f}$ denotes the bond strength at room temperature. The coefficients in Equation (44) are obtained by calibration according to experimental data. Figure 3b compares the experimental results with the predicted values.

**Figure 4:**
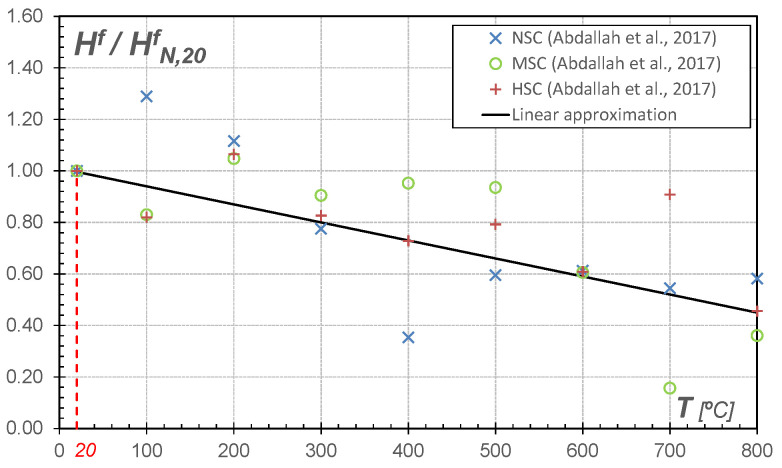
Normalized temperature-dependent pull-out softening parameter estimation based on the experimental results reported by [21].

Once $\sigma_{y}^{f}$ is reached, the comparison stress undergoes linear softening according to the evolution of the internal variable ${\dot{\kappa }}_{N}^{f}$ and proportionally to the softening parameter $H_{N}^{f}\left(T\right)$. In order to reproduce the increase in ductility associated with the rise in fiber temperature, a normalized linear approximation is proposed as

(45) $$\frac{H_{N,T}^{f}}{H_{N,20}^{f}}=A_{H}\;T+B_{H}\;,$$

where $H_{N,20}^{f}$ represents the softening modulus at room temperature and the coefficients $A_{H}$ and $B_{H}$ are calibrated using experimental data. Figure 4 compares the numerical predictions obtained from Equation (45) with results from the experimental campaign by Abdallah et al. [21].

### 6.2. Effect of High Temperature on the Steel Fiber Dowel Mechanism

Although it has been demonstrated that the addition of fibers mitigates the reduction in shear strength even at high temperatures [15,16], no experimental studies have specifically investigated the dowel behavior of individual fibers bridging cracks.

Assuming that thermal degradation of shear strength follows a similar trend to that observed for the bond-slip degradation case, the same criteria adopted in the previous section are applied herein. It should be noted that the proposed degradation law for the dowel contribution is introduced as a phenomenological assumption motivated by the analogous deterioration observed in bond-slip mechanisms at elevated temperatures. Therefore, the formulation should be interpreted within the scope of residual mechanical response analyses after exposure to high temperatures validated in the present work. Additional experimental studies specifically focused on the dowel behavior of individual fibers after thermal exposure, which would be required to establish more physically based degradation relationships. Under these assumptions, the evolution of the shear stiffness $G^{f}\left(T\right)$, the equivalent strength $\tau_{y}^{f}\left(T\right)$ and the softening parameter $H_{T}^{f}\left(T\right)$, are described by decay functions analogous to Equations (43)–(45), respectively. It should be recalled that, at room temperature, $G_{f}$ and $\tau_{y}^{f}$ are derived from the definition of the stiffness and strength of a generic fiber embedded in a concrete matrix and subjected to a transverse force. This formulation is based on the analogy with a *semi-infinite* beam on a Winkler foundation, following the contributions by Soroushian and Lee [46], Dei P. et al. [52], Dulacska [53], El-Ariss [54].

The main constitutive laws corresponding to the dowel behavior of fibers crossing cracks are listed in the third column of Table 1. The constitutive equation is based on the Prandtl–Reuss additive decomposition of tangential strains, $\varepsilon_{T}^{f}=\varepsilon_{T}^{f,e}+\varepsilon_{T}^{f,p}$, and on the temperature-dependent shear stiffness $G^{f}\left(T\right)$. In addition, the comparison stress of the yield criterion is composed of the equivalent strength $\tau_{y}^{f}\left(T\right)$ and the dissipative stress $\phi_{T}^{f}$, which depends on the softening parameter $H_{T}^{f}\left(T\right)$ and its conjugated internal variable $\kappa_{T}^{f}$.

## 7. Numerical Solutions for Localized Failure in Microplane-Based Elasto-Plasticity

This section presents the main features and capabilities of the proposed formulation at material level to predict the fracture behavior of SFRC after exposure to high temperatures [55]. Although the constitutive framework presented in Section 2 is formulated within a general thermo-mechanical setting, including thermal gradients, heat flux and coupling effects, the numerical simulations reported herein are restricted to the residual mechanical response after thermal exposure. Accordingly, the analyses are performed under isothermal conditions after the preheating stage, while the effect of elevated temperatures is incorporated through the temperature-dependent degradation of the constitutive parameters and internal variables associated with the maximum attained temperature. With this aim, the thermodynamically consistent elastoplastic microplane model, together with the corresponding algorithmic tangent operator defined in Equation (17), was implemented within the framework of mixture theory. Furthermore, the two-dimensional microplane formulation proposed by Park and Kim [35] was adopted instead of the spherical one.

The combined effects of added steel fibers and high preheating temperatures on the failure behavior are further evaluated by assessing the critical condition for localized failure in the form of discontinuous bifurcation, as explained in Section 3, whereas the diffuse failure condition agrees with the peak load.

To demonstrate the robustness of the proposed formulation, two types of tests that activate both Modes I and II of failure were selected: uniaxial tensile and direct shear tests, respectively.

**Figure 5:**
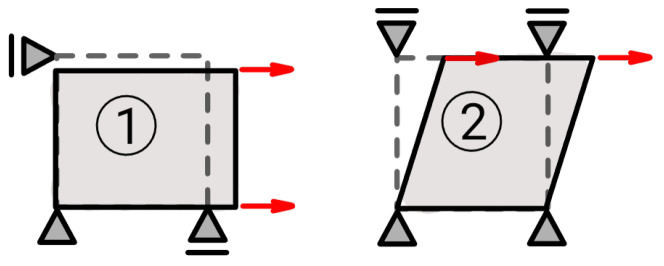
Adopted load and boundary configurations for (1) uniaxial tensile and (2) direct shear tests. Arrows indicate the applied loading direction.

The numerical results are compared with experimental data available in the literature, in terms of stress–crack opening displacement (COD) (or crack slip) curves. In both load cases, the contribution of reinforcement becomes particularly relevant. Compression tests are not considered here, since the contribution of fibers under compression is generally less significant than under tensile or fracture-dominated conditions, as widely reported in the literature [12,17,18,19]. The stress–COD (or crack slip) responses were obtained by means of finite element routines developed by the authors, using a single finite element with four Gauss integration points. Adopted load and boundary configurations for each case are shown in Figure 5. Material properties reported by the corresponding experimental studies are summarized in Table 2, whereas Table 3 compiles the constitutive model parameters adopted or calibrated from experimental data.

In order to verify the ability of the proposed constitutive model to predict uniaxial tensile states, the experimental results of the splitting tensile (ST) test performed by Xargay et al. [11] have been used as a reference. Although the splitting tensile (ST) test does not reproduce a strictly uniform uniaxial tensile stress state, it is widely used to characterize the tensile fracture response of fiber-reinforced concretes due to the formation of a dominant crack plane governed by tensile stresses. In the present study, the proposed formulation is intended to reproduce the residual tensile transfer mechanisms associated with fiber bridging after thermal exposure, which can be indirectly assessed through the ST experimental response reported by Xargay et al. [11]. This experimental campaign was carried out on cylindrical specimens of 100 mm in diameter and 200 mm in height, made of plain self-compacting high-strength concrete (SCHSC) and self-compacting fiber-reinforced high-strength concrete (SCFRHSC), containing 0.76% steel fiber by volume. The corresponding material properties are listed in the second column of Table 2. Two temperatures were considered in the heating phase, namely 300 °C and 600 °C, in addition to the room temperature case. The experiments were performed under residual conditions.

### 7.1. Uniaxial Tensile Test

**Figure 6:**
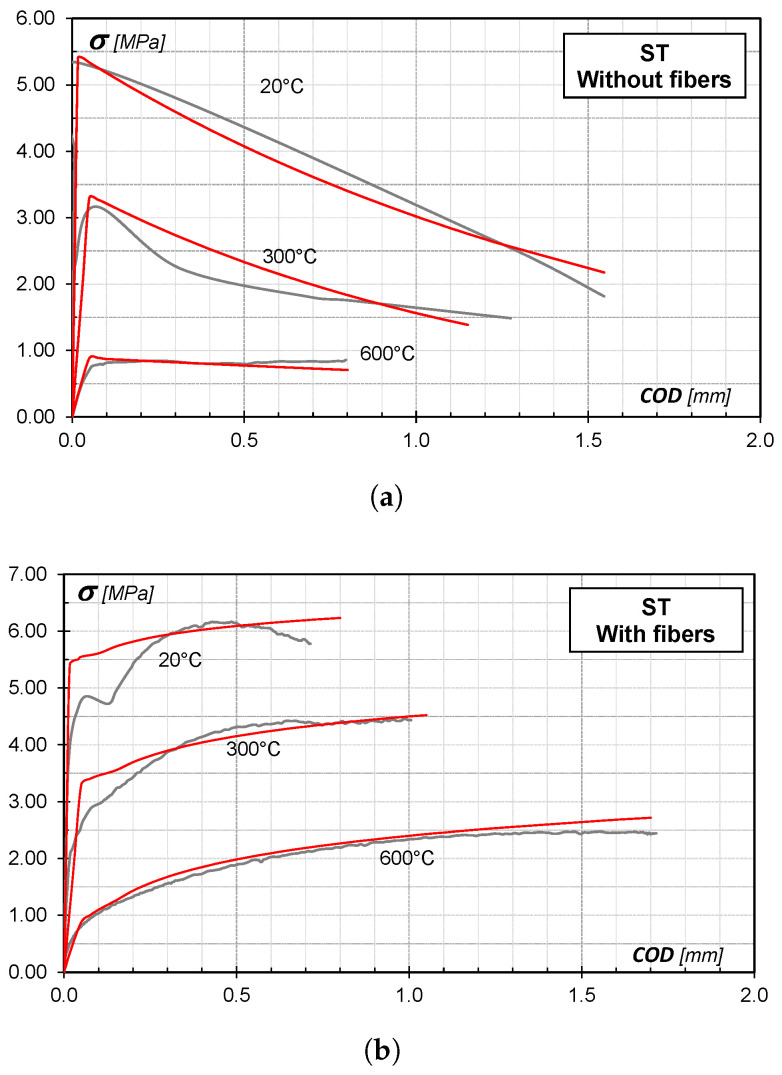
ST test: Mean tensile stress versus COD at different temperatures in (**a**) Plain concrete and (**b**) SFRC. Red lines represent the numerical model predictions, whereas gray lines correspond to the experimental curves.

Figure 6 compares the numerical responses against the experimental ones in terms of mean tensile stress versus COD, showing acceptable agreement. The results indicate that the addition of fibers to the cementitious matrix improves both the tensile strength and the fracture energy dissipation capacity of the concrete, not only under room-temperature conditions, but also at moderate and high temperatures.

For the preheated specimens, a significant degradation of strength is observed as the temperature increases, which is more pronounced in the case of plain concrete. This behavior can be explained by the more tortuous crack paths observed in the samples exposed to higher temperatures. Nevertheless, the fiber-reinforced specimens maintain their structural integrity.

**Figure 7:**
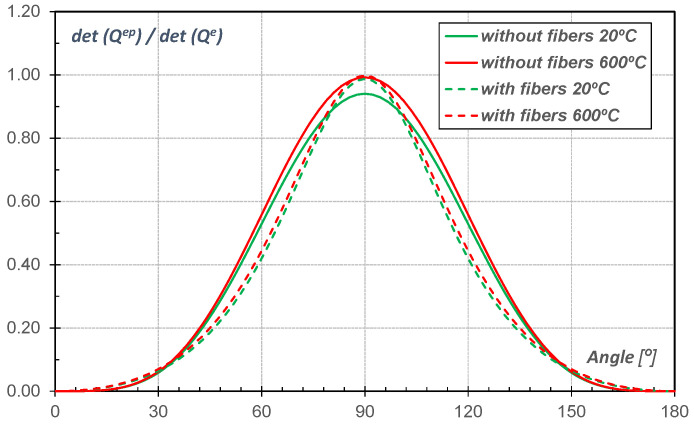
Numerical localized failure analysis at peak of the ST test.

The performance of the discontinuous bifurcation condition in the form of localized failure given in Equation (25) is useful for detecting critical failure directions, which, in the case of uniaxial tension, results in 90° for plain and reinforced concrete, whatever the considered temperature. The localization analysis shown in Figure 7 demonstrates that for plain concrete at 20 °C, the failure behavior is highly brittle and unstable, whereas the fiber-reinforced specimens exhibit a more stable response consistent with the observed structural integrity.

### 7.2. Shear Test

The influence of steel fibers on the shear behavior of concrete at different temperatures was experimentally investigated by Alimrani and Balazs [15]. In that study, push-off specimens made of plain and steel fiber-reinforced concretes (0.5% and 1% fibers by volume) were tested at 20 °C and after preheating to 500 °C.

**Figure 8:**
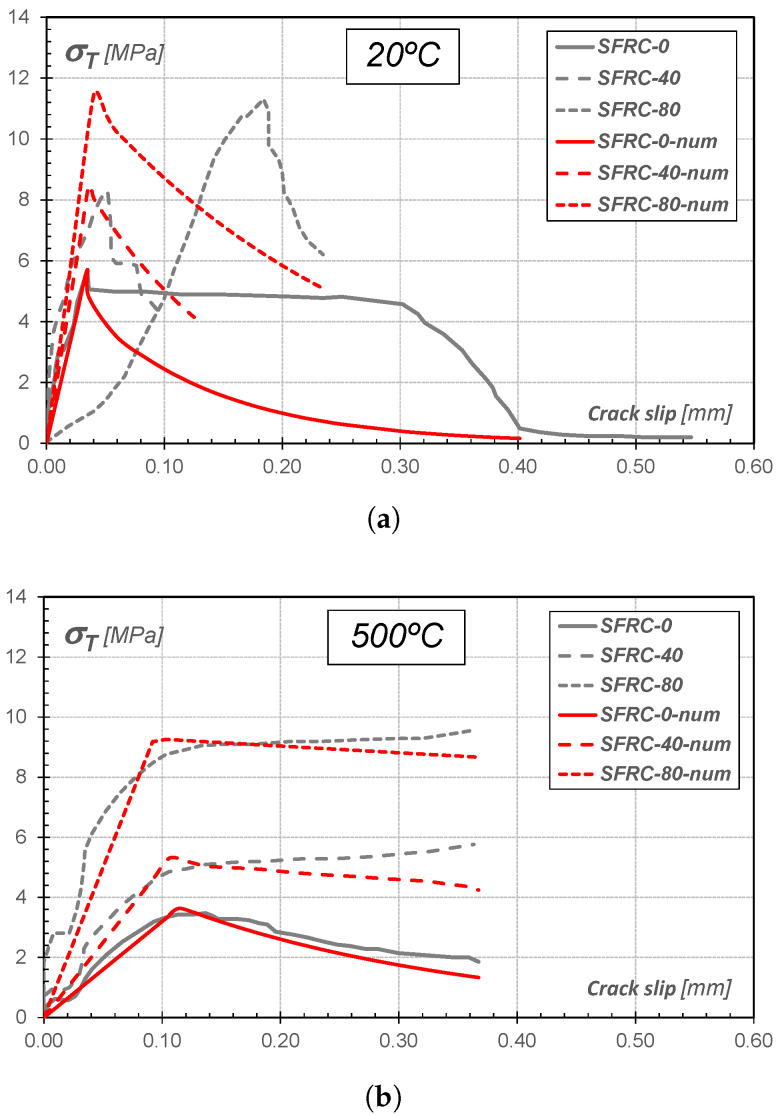
Shear test: Shear stress versus crack slip curves for different fiber content at (**a**) 20 °C and (**b**) 500 °C.

As can be seen in Figure 8, the presence of steel fiber increases both the shear strength and ductility of concrete in room-temperature conditions as well as after exposure to elevated temperatures.

Regarding the numerical predictions at 20 °C, good agreement is observed in terms of peak load for all fiber contents. However, the model does not capture the peak crack slip for concrete reinforced with 1% fibers, nor the post-peak fracture energy of plain concrete. In contrast, a better approximation is achieved for concrete reinforced with 0.5% fibers, where the experimental results exhibit greater consistency with the expected behavior, as can be observed Figure 8a.

The mechanical degradation of preheated specimens is evident in Figure 8b, where the mean test results are shown in terms of shear stress versus crack slip. The numerical responses are superimposed, demonstrating good agreement with the experimental results.

While fiber-free specimens exhibit a clear shear failure, which becomes more pronounced when preheated to very high temperatures, fiber-reinforced specimens show a significantly different failure mode that appears to be diffuse. The presence of fibers controls crack opening, preventing the complete splitting of the specimen. Moreover, surface spalling is observed in the region surrounding the shear plane. At very high temperatures, the effectiveness of steel fibers decreases due to thermal degradation, allowing larger crack openings to develop.

**Figure 9:**
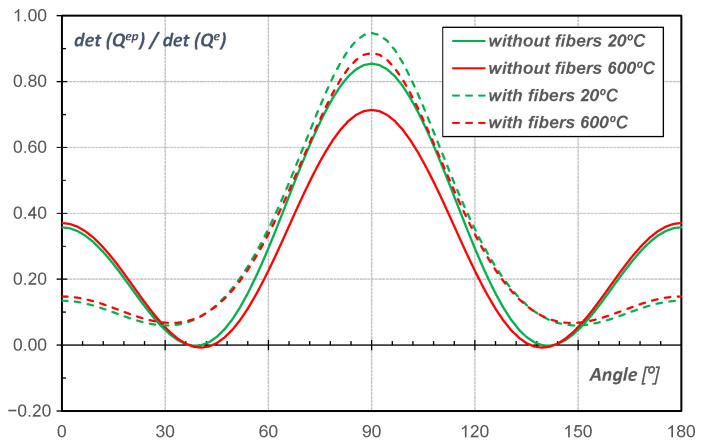
Numerical localized failure analysis at peak of the shear test.

The performance of the determinant of the normalized macroscopic localization indicator regarding the contribution of fibers and preheating temperature is evaluated in Figure 9. The obtained critical angles are consistent with the observed failure modes. Plain concrete specimens exhibit almost vertical and straight failure planes, whereas fiber-reinforced specimens develop more inclined and tortuous crack patterns. As the temperature increases, the failure plane in plain concrete remains essentially vertical, while in fiber-reinforced concrete it becomes progressively more inclined. For specimens exposed to temperatures close to 600 °C, the failure pattern becomes more distributed and tortuous in both cases, although the fiber-reinforced specimens exhibit less severe damage.

The numerical solutions for discontinuous bifurcation in temperature-dependent media presented in this work offer significant potential for more accurate predictions and improved understanding of concrete failure modes under different thermo-mechanical scenarios.

## 8. Conclusions

In this work, a traditional thermodynamically consistent microplane constitutive model for quasi-brittle materials such as SFRC has been extended to consider the effects of high-temperature fields. The well-known mixture theory has been applied to address the composite behavior, accounting the cementitious matrix and micromechanical fiber–matrix interactions phenomena, i.e., axial bond-slip and tangential dowel effect.

The new formulation incorporates the dependency of the elastic and mechanical concrete properties on the temperature as well as the fiber–matrix interactions deterioration, through degradation functions based on experimental evidence.

Numerical simulations performed for uniaxial tensile and direct shear tests show that the numerical model is able to capture the main features of the failure process after preheating, i.e., the degradation of strengths, which is more pronounced in the case of plain concrete, and the improvement of the post-peak ductility with the addition of fibers.

Analytical solutions for the acoustic tensor for temperature-dependent microplane-based models have been derived, and numerical evaluations of the acoustic tensor have allowed the determination of its critical directions at the peak load.

In case of tension, a more stable response is observed with the addition of reinforcement, although the critical angles remain unchanged. In case of direct shear, an increase in the slope of the failure plane is predicted.

Future developments will address the incorporation of directional temperature effects in combination with predominant fiber orientations. Also, future research will focus on extending the validation of the proposed formulation to larger-scale specimens, i.e., three-point bending (TPB) tests and structural elements subjected to thermal exposure, including comparisons with dedicated experimental programs. Additional constitutive benchmark assessments, such as Willam-type single-element tests under multiaxial loading paths, will also be considered to further evaluate the predictive capabilities of the model. In particular, prescribed displacement paths in Willam-type tests could provide an integrative assessment combining both bond-slip and dowel-effect mechanisms.

## Figures and Tables

**Table 1:** Constitutive laws for fiber bond-slip and dowel effect under high temperatures.

	1D Bond-Slip Model	1D Dowel Model
Constitutive equation	$\sigma_{N}^{f}=E^{f}\left(T\right)\;\left(\varepsilon_{N}^{f}-\varepsilon_{N}^{f,p}\right)$	$\sigma_{T}^{f}=G^{f}\left(T\right)\;\left(\varepsilon_{T}^{f}-\varepsilon_{T}^{f,p}\right)$
Yield condition	$\Phi_{N}^{f}=\left|\sigma_{N}^{f}\right|-\left(\sigma_{y}^{f}\left(T\right)+\phi_{N}^{f}\right)\leq 0$	$\Phi_{T}^{f}=\left|\sigma_{T}^{f}\right|-\left(\tau_{y}^{f}\left(T\right)+\phi_{T}^{f}\right)\leq \;0$
Internal variable evolution	${\dot{\kappa }}_{N}^{f}=\dot{\lambda }$	${\dot{\kappa }}_{T}^{f}=\dot{\lambda }$
Softening law	${\dot{\phi }}_{N}^{f}=H_{N}^{f}\left(T\right)\;{\dot{\kappa }}_{N}^{f}$	${\dot{\phi }}_{T}^{f}=H_{T}^{f}\left(T\right)\;{\dot{\kappa }}_{T}^{f}$

**Table 2:** Material properties.

Tests	Uniaxial Tensile Xargay et al. [11]	Direct Shear Alimrani and Balazs [15]
Concrete		
$E^{m}$ [GPa]	40.0	40.0
ν	0.2	0.2
$f_{c}^{\prime }$ [MPa]	80.0	78.83
$\sigma_{dil}$ [MPa]	16.0	16.0
Steel fibers		
$E^{d}$ [GPa]	200.0	200.0
$\sigma_{y}^{d}$ [MPa]	1100.0	2300.0
$G^{f}$ [GPa]	15.0	30.0
$\tau_{y}^{f}$ [MPa]	330.0	700.0

**Table 3:** Constitutive model parameters.

Parameter	Value	Description
Concrete		
$\alpha_{E}$	0.0014	Calibrated in [48]
$\alpha_{\nu }$	0.0010	Calibrated in [48]
$\gamma_{1}$	0.0025	Calibrated in this work
$\gamma_{2}$	0.0011	Calibrated in this work
$A_{h}$	−0.00308	Calibrated in this work
$A_{G}$	−0.000004	Calibrated in this work
$B_{G}$	0.0027	Calibrated in this work
$C_{G}$	0.95	Calibrated in this work
Steel fibers		
$A_{H}$	−0.0007	Calibrated from [21]
$B_{H}$	1.00	Calibrated from [21]
$H_{N}^{f}$ = $H_{T}^{f}$	0.0	Adopted in this work
Fiber-concrete interfaces		
$E^{f}$ [GPa]	200.0	Calibrated in this work
$\sigma_{y}^{f}$ [MPa]	210.0	Calibrated in this work
$A_{E}$	−0.0009	Calibrated from [21]
$B_{E}$	1.0224	Calibrated from [21]
$A_{\sigma }$	−0.0009	Calibrated from [19,21]
$B_{\sigma }$	1.018	Calibrated from [19,21]

## Data Availability

The original contributions presented in this study are included in the article. Further inquiries can be directed to the corresponding author.
